# Genomic Background and Phylogeny of *cfi*A-Positive *Bacteroides fragilis* Strains Resistant to Meropenem-EDTA

**DOI:** 10.3390/antibiotics10030304

**Published:** 2021-03-16

**Authors:** Sylvia Valdezate, Fernando Cobo, Sara Monzón, María J. Medina-Pascual, Ángel Zaballos, Isabel Cuesta, Silvia Pino-Rosa, Pilar Villalón

**Affiliations:** 1 National Centre of Microbiology, Reference and Research Laboratory for Taxonomy, Instituto de Salud Carlos III, Majadahonda, 280220 Madrid, Spain; mjmedina@isciii.es (M.J.M.-P.); silvia.pino@isciii.es (S.P.-R.); pvillalon@isciii.es (P.V.); 2 Department of Microbiology, Instituto Biosanitario de Granada, University Hospital of Virgen de las Nieves, Avda. Fuerzas Armadas s/n, 18014 Granada, Spain; fernando.cobo.sspa@juntadeandalucia.es (F.C.); smonzon@isciii.es (S.M.); i.cuesta@isciii.es (I.C.); 3 Bionformatics Unit, Applied Services, Training and Research, Instituto de Salud Carlos III, Majadahonda, 280220 Madrid, Spain; azaballos@isciii.es; 4 Genomics Unit, Applied Services, Training and Research, Instituto de Salud Carlos III, Majadahonda, 280220 Madrid, Spain

**Keywords:** *Bacteroides fragilis*, susceptibility, multidrug resistance, *cfi*A14, *cfi*A28, division II, carbapenems, porin inactivation

## Abstract

Background: *Bacteroides fragilis* shows high antimicrobial resistance (AMR) rates and possesses numerous AMR mechanisms. Its carbapenem-resistant strains (metallo-β-lactamase *cfi*A-positive) appear as an emergent, evolving clade. Methods: This work examines the genomes, taxonomy, and phylogenetic relationships with respect to other *B. fragilis* genomes of two *B. fragilis* strains (CNM20180471 and CNM20200206) resistant to meropenem+EDTA and other antimicrobial agents. Results: Both strains possessed *cfi*A genes (*cfi*A14b and the new *cfi*A28), along with other AMR mechanisms. The presence of other efflux-pump genes, *mex*AB/*mex*JK/*mex*XY-*opr*M, *acr*EF/*mdt*EF-*tol*C, and especially *cus*R, which reduces the entry of carbapenem via the repression of porin OprD, may be related to meropenem–EDTA resistance. None of the detected insertion sequences were located upstream of *cfi*A. The genomes of these and other *B. fragilis* strains that clustered together in phylogenetic analyses did not meet the condition of >95% average nucleotide/amino acid identity, or >70% in silico genome-to-genome hybridization similarity, to be deemed members of the same species, although <1% difference in the genomic G+C content was seen with respect to the reference genome *B. fragilis* NCTC 9343T. Conclusions: Carbapenem-resistant strains may be considered a distinct clonal entity, and their surveillance is recommended given the ease with which they appear to acquire AMR.

## 1. Introduction

*Bacteroides fragilis* is a common bacterium of the human gut. It generally behaves as a commensal species, but under certain conditions it can cause severe intra-abdominal infections, skin and soft tissue infections, brain abscesses, surgical site infections, and anaerobic bacteraemia [1]. The plasticity of the *B. fragilis* genome allows it to incorporate antimicrobial resistance and virulence determinants via horizontal gene transfer (HGT), and to turn specific resistance genes on or off as needed [2,3]. Its behavior as a reservoir of resistance is well-established [1,2,3].

Antimicrobial resistance surveys have reported on *B. fragilis* populations to show high resistance (rates > 80%) to penicillins, cephalosporins, and tetracyclines; medium resistance (5–35%) to β-lactam/β-lactamase inhibitor combinations, cephamycins, macrolide-lincosamide-streptogramin B (MLSB) drugs and fluoroquinolones; and low resistance (<5%) to carbapenems, metronidazole, and tigecycline [4]. Around 2–8% of *B. fragilis* strains are resistant to carbapenems (just 1–4% in Spain) in Europe and its neighboring countries [5,6,7]. The main antimicrobial resistance (AMR) determinants involved are *cep*A for penicillins and cephalosporins, *cfx*A for cephamycins, *cfi*A for carbapenems, *erm*F for MLSB compounds, *nim*A–J for metronidazole, *tet*Q for tetracyclines, and *gyr*A point mutations and *bex*A/B (coding for efflux pumps) for fluoroquinolones [4,8,9,10]. Some of these traits are commonly encoded by chromosomal genes carried on integrative and conjugative elements (ICEs), including conjugative transposons (CTns), compound transposons, and mobilizable plasmids [2,3,9,10,11,12].

The *B. fragilis* population can be separated into two divisions, I and II, depending on the mutually exclusive presence of the chromosomal cephalosporinase gene *cep*A or the chromosomal metallo-β-lactamase (MBL) gene *cfi*A, together with other characteristics [4,13,14]. *cfi*A confers a high level of resistance to carbapenems but also to other β-lactams and β-lactamase inhibitor combinations. In addition, the expression levels of *cfi*A, *nim*, and *erm* (providing resistance to metronidazole and macrolides, respectively) are partly controlled by upstream insertion sequences (IS) found in multiple copies throughout *B. fragilis* genomes [3,10].

The aim of the present study was to characterize two *cfi*A-positive *B. fragilis* strains resistant to meropenem+EDTA. The phenotypic detection of MBLs in *B. fragilis* is usually undertaken using the meropenem-EDTA double-ended Etest [15]. However, the studied strains were resistant to this, and the presence of *cfi*A could not be inferred.

## 2. Results and Discussion

### 2.1. Species Identification and Susceptibility Phenotype

*B. fragilis* CNM20180471 and CNM20200206 strains were initially identified by MALDI-TOF/MS as *B. fragilis* with a score of 2.11 and 2.38 (mean of duplicates), respectively. They were confirmed by 16S rDNA analysis: BLAST examinations returned 99.01% and 98.75% identities with respect to the sequence of the type strain *B. fragilis* NCTC 9343T (GenBank accession no. KP326374.1) [16]. According to EUCAST and CLSI criteria, the strains showed resistance to amoxicillin-clavulanate, piperacillin-tazobactam, carbapenems, aminoglycosides, fluoroquinolones, erythromycin, clindamycin, and tetracycline; they were susceptible to metronidazole, linezolid, and tigecycline (Table 1). No differences in categorization were detected between both criteria. No MBL activity was detected for either strain by the meropenem double-ended Etest (Figure S1); no reduction in the MIC of meropenem was seen when EDTA was present. However, MBL activity was detected when imipenem and EDTA were present. The last combination was more effective in detecting these MBLs than the recommended meropenem double-ended Etest in the studied strains [15].

### 2.2. Susceptibility Genotype

Two different metallo-β-lactamases were involved in the carbapenem resistance of these strains. In the CNM20180471 strain, the metallo-β-lactamase *cfi*A14b, fully identical to that of the carbapenem-resistance protein (*cfi*A) in *B. fragilis* K1I3 (GenBank accession no. KT318729.1), was detected [17]. In the CNM20200206 strain, a newly assigned metallo-β-lactamase *cfi*A28 (GenBank accession no. MBE7399711.1, pd-help@ncbi.nlm.nih.gov) similar to *cfi*A-23 and *cfi*A-27 (99.6% identity, with the substitutions 113-Arg and 188-Ala, respectively; GenBank accession nos. WP_085562386 and AUW27645.1, respectively) [17], and identical to that in *B. fragilis* HMW 610 (GenBank accession no EKA90191.1), was identified. In both strains, other AMR traits (identity ≥ 98%) were seen (Table 1). In the CNM20180471 strain, *mef*(En2) and *lnu*(AN2) genes were contiguous, as were *cfi*A and *qac*E genes. Other resistance traits identified in *B. fragilis* by other authors, such as the production of beta-lactamases (*cep*A, *cfx*, *bla*-OXA), of the methyltransferase *erm*B and *erm*G, and of the efflux pump msrSA involved in macrolide-lincosamide-streptogramin (MLS) resistance [18], were not detected in the present strains. Neither were nitroimidazole resistance genes (*nim* A-H/J/L) detected, in agreement with their susceptibility to metronidazole.

A clear pheno-to-genotype correlation was found for strain CNM20180471, with the following exceptions: (i) the lack of an effect of the combination meropenem–EDTA on MBL activity despite the presence of *cfi*A; (ii) high fluoroquinolone MICs despite the conserved *gyr*A and *gyr*B genes (with motifs Asp81-Ser82-Gly478 and Leu415, respectively), explained by the presence of fluoroquinolone-resistant, active multidrug and toxic compound extrusion (MATE) efflux-pumps, *bex*A/*bex*B [19]; (iii) and susceptibility to tigecycline despite the presence of *tet*X [20]. The first two exceptions (i) and (ii) were also detected for strain CNM20200206, together with high resistance to macrolide-lincosamide-streptogramin compounds, with no detection of the genes involved.

KEGG orthology, used to search for gene set resistance modules, detected *cus*R in both strains, which is a phosphate regulon response regulator of the two-component system of the OmpR family providing copper resistance. *cus*R reduces the entry of carbapenems via the repression of the porin OprD, thus affording imipenem resistance [21]. The MBLs *cfi*A14b and *cfi*A28 were inhibited by EDTA when combined with imipenem but also with meropenem. In the studied strains, the exact mechanism underlying the lack of susceptibility to meropenem-EDTA was not detected on the basis of functional annotation, but the above finding of *cus*R may be related to a reduction in meropenem permeation, perhaps due to the inactivation of a porin different from OprD. In both strains, other gene set resistance modules found were linked to resistance to vancomycin (*van*X), daptomycin (*dlt*A), and multidrug resistance efflux pumps (*mex*AB-*opr*M, *mex*JK-*opr*M, *mex*XY-*opr*M, *acr*EF-*tol*C, and *mdt*EF-*tol*C). The operon identified as *mex*AB-*opr*M is equivalent to *bme*ABC for the resistance-nodulation-division RND efflux pumps described in *B. fragilis* (involved in beta-lactam resistance). Efflux pumps have an important role in multiresistance in *B. fragilis* [22]. Exposure to several beta-lactams, carbapenems, fluoroquinolones, metronidazole, and certain chemical agents all induce the over-expression of efflux pump genes, thus promoting MDR in *B. fragilis* [23]. Other regulators of multiple antibiotic resistance such as the MarR family may be involved, as recently seen when the abolishment of the expression of MarR homologs increased susceptibility to several antimicrobials in *B. fragilis* [24]. The MarR sequences of the studied strains showed full identity, as they did with those of strains O21, BF8, and DCMSKEJBY0001B [4]. The interplay of the efflux pumps and the regulators of multiple antibiotic resistance, together with changes in porins, may be responsible for the lack of effect of the meropenem-EDTA combination in the studied strains, which may be another step in the evolution of the emergent clade of the carbapenem-resistant *B. fragilis* strains.

### 2.3. IS Elements

Five IS types were identified in strain CNM20180471: (i) *IS*612B (a copy number of one, an identity with respect to platform references of 100%, and a length coverage of 100%), which is highly homologous to *IS*612 and *IS*614; the latter two activate the *cfi* carbapenemase genes in some *B. fragilis* strains [10,19]; (ii) *IS*Baov1 (1, 100%, 100%) which is described as inserted in conjugative transposon CTnGERM1 of *Bacteroides ovatus* [11]; (iii) *IS*Bf3 (one full copy and two partial copies, 99.8%, 95.6–100%), detected in plasmid pBFY46; (iv) *IS*Bf5 (1, 99.7%, 96.1%), detected in the genome of *B. fragilis* YCH46 [25]; (v) and *IS*Bf9 (3, 86–99%, 30–71%; plus eight shorter copies). In strain CNM20180471, *IS*Baov1 was located upstream of *erm*F (contigs = 04264 and 04267, respectively) [11]. However, *IS*612B was located together with *IS*Bf3 and *IS*Bf5 (contigs = 04256, 04252, and 04253, respectively) far away from *cfi*A14b (contig = 01848).

In CNM20200206, three IS types were identified: (i) *IS*4351 (a copy number of one, an identity of 100%, and a coverage of 100%), which appeared to be closely related to *IS*4400 on Tn4400 of *B. fragilis* [26]; (ii) *IS*Bf3 (1, 100%, 100%); (iii) and *IS*Bf9 (1, 99%, 71%; plus three shorter copies). In this strain, *IS*4351 was located together with *IS*Bf9 (contigs = 04535 and 04534). None of these *IS* were located upstream of the new *cfi*A28 gene. The expression level of antimicrobial resistance is partly controlled by *IS*s carrying a promoter sequence upstream of the *cfi*A genes [10,27]. Different *IS*s (IS612, -613, -614, -615, -942, -943, -1186, -1187, and Bf12) can act as efficient promoters of *cfi*A in *B. fragilis*, although sometimes no *IS* appears upstream of the gene [10,12], as seen for both strains. These results are summarized in Table S1.

### 2.4. Plasmids

In *B. fragilis*, the AMR genes are spread across conjugative and mobilizable plasmids, and across integrative genetic elements, including conjugative transposons [2,3,28]. No hits were detected by PlasmidFinder-2.0 for both strains, but the pLSDB (a plasmid database) server revealed the CNM20180471 genome to strongly match: (i) *B. fragilis* plasmid Q1F2-p1 belonging to the pBSSB1 family (GenBank accession no. NZ_CP018938.1, size ~4.5 Kb) (identity = 99.12%); (ii) the *B. fragilis* mobilizable plasmid pBFUK1 of the IncA/C family (NC_019534.1, ~13 Kb), which carries the metallo-β-lactamase *cfi*A [12] and *IS*613/Baov1 (identity = 95.01%); and (iii) the *Bacteroides thetaiotaomicron* plasmid p2-F9-2 (AP022662.1, ~0.8 Kb) of the IncA/C family, which carries the *tet*X gene (identity = 92.64%). The CNM20200206 genome also showed a strong match with plasmids Q1F2-p1 and pBFUK1 (identities = 98.42% and 94.09%, respectively) and pBFY46 plasmid (NC_006297.1, ~34 Kb, 86.45%) (Table S1).

A putative integrative conjugative element INT_ICEBs1_C_like (C-terminal catalytic domain of integrases from bacterial phages and conjugate transposons) was also annotated in CNM20180471 (contig = 01286) [2], and was identical to the nucleotide sequence of a site-integrase tyrosine recombinase XerD type of *B. fragilis* DCMOUH0067B (GenBank accession no. CP036553) [10]. *B. fragilis* acquires and efficiently disseminates traits of its pan-genome (AMR, virulence, and the rapid adaptation to a changing environment) via conjugative and mobilizable elements [2,3].

### 2.5. Genome-Based Taxonomy and B. fragilis Phylogeny

For strain CNM20180471, a genome length of 5,070,925 bp (138 contigs, N50: 140,777), with a 43.20% G+C content, 4292 genes (4229 protein-coding genes), 3 rRNAs, 59 tRNAs, and 1 transfer-messenger RNA (tmRNA), were predicted by Prokka software. For strain CNM20200206, the values were a length of 5,532,196 bp (111 contigs, N50: 86,235), a 43.65% G+C content, with 4358 genes (4481 protein-coding genes), 3 rRNAs, 53 tRNAs, and 1 transfer-messenger RNA.

Although the difference in the G+C content was <1% (Table S1), the average nucleotide identity (ANI) and average amino acid identity (AAI) percentage values and the *in silico* genome-to-genome distance similarity (GGDH) or DNA–DNA hybridization (DDH) -estimate for CNM20180471 and CNM20200206 with respect to the genomes of *B. fragilis* NCTC 9343T (86.96, 90.07, 32.8 and 87.23, 90.29, 33.10, respectively) and *B. fragilis* YCH46 (87.12, 90.5, 33 and 87.00, 90.21, 33.30, respectively) suggested the studied strains belong to different species (<95% ANI/AAI, <70% DDH-estimate) [29,30]. Between strains CNM20180471 and CNM20200206, these coefficients reached values of 98.54 and 97.11 and 85.80 respectively.

**Figure 1:**
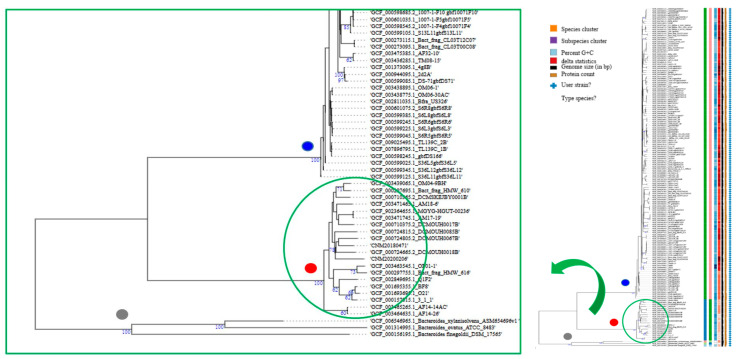
*B. fragilis **w***hole-genome, sequence-based phylogenetic tree constructed using FastME v.2.1.6.1 software (which calculates genome BLAST distance phylogeny (GBDP) distances); the branch lengths are scaled in terms of the GBDP distance formula. The numbers above the branches are GBDP pseudo-bootstrap support values (all >60%; 100 replicates); average branch support = 27.9%. The trees were rooted at the midpoint. The results were provided by the Type Strain Genome Server (TYGS) (https://tygs.dsmz.de, accessed on 19 October 2020) [33]. The same color code was used in the chewBBACA analysis.

**Figure 2:**
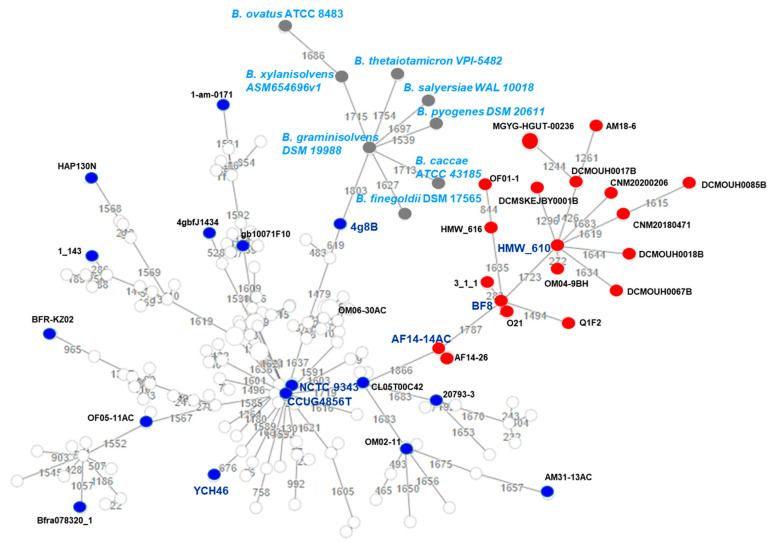
Phylogenetic tree (constructed by the multiple sequence alignment (MAFFT) and neighbor joining using the Clustal W2 algorithm) based on the core genome Multilocus Sequence Type associations among the *B. fragilis* genomes and those of the related examined species. The tree was built using chewBBACA software [34] and was based on 2362 loci. The NCTC 9343^T^ (NC_003228.3) and the CCUG4856 (GCF_005706655.1) genomes correspond to the *B. fragilis* type strain, while YCH46 (NC_006347.1) is the representative genome. Genomes with red balls correspond to genomes with an AAI value of <88% with respect to strain NCTC 9343^T^; genomes with blue balls correspond to genomes with an AAI value of ~99% with respect to the same strain; genomes with grey balls correspond to genomes of other *Bacteroides* species that have an AAI value of ~75% with respect to NCTC 9343^T^. Branches indicate the number of different alleles.

TYPS analysis [31] returned two different clusters: a main one that grouped 87.7% of the *B. fragilis* genomes and a smaller one encompassing 12.3% of the strains; CNM20180471 and CN20200206 fell into this latter cluster with 18 other *B. fragilis* genomes (Figure 1). The same clustering was observed with the chewBBACA (BSR-Based Allele Calling Algorithm) minimum spanning tree (MST) [32] (Figure 2; blue balls for the larger cluster and red balls for the smaller cluster). The node of this cluster is represented by the HMW_610 genome (GCF_000297695.1) [2]. The genomes of strains AF14-14AC (GCF_003465265.1) and 4g8B (assembly no. GCF_001373095) were located at intermediate points between the central node and that occupied by the genome of CCUG 4856T (GCF_005706655.1), analogous to the reference genome *B. fragilis* NCTC 9343T (NC_003228.3) and the HMW_610 genome. 

The ANI, AAI, DDH-estimate, and G+C content difference values were determined with respect to the NCTC 9343T for all the genomes included in the above smaller cluster and for selected genomes of the large cluster. The ANI value was also determined with respect to the YCH46 and CNM20180471 genomes (Table S2). The strains belonging to the smaller cluster had the lowest ANI values with respect to NCTC 9343T and YCH46 (~88%) but not with respect to CNM20180471 (~98.44%). The members of the smaller cluster therefore showed less identity (and coverage) to the type strain than did the members of the larger cluster. The examined strains belonging to the larger cluster returned high ANI values with respect to NCTC 9343T (~99%) and YCH46 (~99%) and lower values with respect to CNM20180471 (~87%). The genomes of the reference strains of other species, such as *Bacteroides finegoldii* DSM 17565 (GCF_000156195.1), *Bacteroides xylanisolvens* ASM654696v1 (GCF_006546965.1), and *Bacteroides ovatus* ATCC 8483 (GCF_001314995.1), showed ANI/AAI values of around 75% with respect to the NCTC 9343T and YCH46 genomes (Table S2).

The ANI, AAI, and DDH-estimate results suggest *B. fragilis* CNM20180471 and CNM20200206, and other members of its cluster with carbapenem-resistance via metallo-β-lactamase *cfi*A, support the previous hypothesis, according to which they are genomospecies “related to, but distinct from, *B. fragilis*” [35] or in a “quasi-transition state between the two divisions of *B. fragilis*” [4]. The same has been stated for carbapenem-resistant *B. fragilis* strains by 16S rRNA analysis (as can be observed in Figure S2), without the bias of their resistome in the whole-genome analysis.

Over the last decade, a world-wide increase in these carbapenem-resistant strains has been reported [26,36,37]; they are favored for their efficient pan-genome dissemination [2,38]. Their evolution may be guided by the impact on the gut ecosystem of carbapenems and other β-lactams [2] used to treat infections caused by extended-spectrum β-lactamase-producing *Enterobacteriaceae* as well as exposure to other antimicrobial and chemical agents. The lack of effect of the meropenem and EDTA combination in the studied *B. fragilis* strains may be another step in the evolution of this complex entity.

In conclusion, the loss of susceptibility against meropenem+EDTA in the studied *cfi*A-positive strains may be due to the presence of multiple efflux-pumps such as MexAB/MexJK/MexXY-OprM, and AcrEF/MdtEF-TolC, but may be especially due to CusR, which can reduce meropenem entry by porin inactivation. No IS was seen upstream of the metallo-β-lactamase *cfi*A14b and *cfi*A28 genes responsible for carbapenem resistance. *B. fragilis* CNM20180471 and CNM20200206, and the other carbapenem-resistant strains examined, may be considered distinct species or novel genomospecies. At the least, they are a different and clear clonal entity of the *B. fragilis* group. The surveillance of this clonal lineage is recommended on the basis of whole genome analyses, including the characterization of MBLs, ISs, and plasmids; antimicrobial susceptibilities observed in this study; and previous indications of the lineage’s ability to acquire and spread antimicrobial-resistance traits.

## 3. Materials and Methods

### 3.1. Case Reports

In case no. 1, a 56-year-old woman undergoing chemotherapy plus peripheral blood precursor treatment for renal amyloidosis was admitted to the intensive care unit at the Hospital Virgen de las Nieves (Granada, Spain) because of a rupture of the spleen with hematoma. After splenectomy, bilateral pleural effusion led to symptoms of respiratory distress. Six months later, the patient developed severe polyneuropathy and became dependent on mechanical ventilation before exitus. In case no. 2, a 52-year-old man with myelodysplasia underwent allogenic transplant at the same hospital. Two days later, he suffered diarrhea caused by toxigenic *Clostridium difficile*; he was treated with oral vancomycin and fidexomicin, and he was cured. Eighteen days later, however, he experienced a febrile episode with hemodynamic instability, and was admitted to an intensive care unit where he was successfully treated with meropenem over a period of 10 days. The clinical samples were taken as part of standard patient care and also for the purpose of this study. This study was focused on bacteria and no identifiable human data were used. Therefore, ethical approval was exempted.

### 3.2. Identification and Antimicrobial Susceptibility Testing

*B. fragilis* CNM20180471 and CNM20200206 were isolated from pleural liquid and blood samples of case no. 1 and case no. 2, respectively. Samples were collected depending on the type of infection. *B. fragilis* growths in anaerobiosis cultures with a single morphology were identified by MALDI-TOF-MS (Bruker Biotyper, Billerica, MA, USA) running v. 9 propriety software (8468 msp). Strain identification was performed by 16S rRNA gene sequence analysis, with primers fD1 and rP2 [39] for amplification, and E781 and U1115 for sequencing [33], using BLAST (http://www.ncbi.nlm.nih.gov/BLAST (accessed on 13 October 2020)).

Susceptibility to antimicrobials was examined using E-test gradient strips (Liofilchem, Teramo, Italy) on Brucella blood agar supplemented with haemin and vitamin K1, using an inoculum of 0.5 McFarland according to the manufacturer’s instructions. MICs were determined after 48 h of incubation at 37 °C in an anaerobic atmosphere. The control strains *B. fragilis* ATCC 25285, *Clostridium perfringens* ATCC 13124, and *Peptostreptococcus anaerobius* ATCC 27377 were used for monthly quality control tests. Resistance to carbapenems were further studied using MBL meropenem-EDTA [15] and imipenem-EDTA double-ended strips (bioMérieux). Results were interpreted following EUCAST (http://www.eucast.org/clinical breakpoints/ (accessed on 13 October 2020)) and CLSI criteria [40,41].

### 3.3. Whole Genome Sequencing and de novo Assembly

Genomic DNA was extracted from single subcultured colonies using the QIAamp DNA Mini Kit (Qiagen). Paired-end libraries were prepared using the Nextera-XT DNA Library Preparation Kit (Illumina 1.9) and sequencing was performed using the Illumina NextSeq 500 High platform (output flow cell ran at 2 × 150). The mean depth of coverage was 243 and 26 for the CNM20180471 and CNM 20200206 strains, respectively.

FastQC v. 0.11.8 software was used to read quality metrics, Trimmomatic v. 0.33 to remove adapter contamination and to trim low quality regions (http://www.usadellab.org/cms/?page=trimmomatic) [42], Kmerfinder v. 3.0 for species confirmation and the detection of contamination (https://bitbucket.org/genomicepidemiology/kmerfinder/src) [43], Spades v. 3.8.0 for de novo assembly (https://cab.spbu.ru/software/spades/) [44], Quast v. 4.1 for assembly quality control (https://github.com/ablab/quast) [45], and Prokka v. 1.12 for genome annotation (https://github.com/tseemann/prokka) (accessed on 13 October 2020) [46].

### 3.4. Antimicrobial Resistance Genes, IS Elements, and Plasmids

AMR genes were detected using the following platforms: srst2 (https://github.com/katholt/srst2) [47], ARGANNOT (https://github.com/katholt/srst2/blob/master/data/ARGannot.fasta) [48], resFinder-4.0 belonging to the Center for Genomic Epidemiology (CGE, database date August 2020, https://cge.cbs.dtu.dk/services/ResFinder/) [49], the Antibiotic Resistant Target Seeker (ARTS, https://arts.ziemertlab.com/) [50], the Comprehensive Antibiotic Resistance Database (CARD, https://card.mcmaster.ca/analyze/rgi) [51], and KOALA for KEGG Orthology (https://www.kegg.jp/ghostkoala/) [52]. The percentage identity threshold for deeming sequences to be equal and the minimum length setting were 90% and 60%, respectively. Those AMR genes not included in databases (*nim*A-J and *bex*A-B) were manually searched for [8]. IS elements were identified using the IS-finder database (https://isfinder.biotoul.fr/) [53]. Plasmid element analysis was performed using the pLSDB (https://ccb-microbe.cs.uni-saarland.de/plsdb/) [34], and the PlasmidFinder-2.0 (CGE, https://cge.cbs.dtu.dk/services/PlasmidFinder/) [54] tools (accessed on 3 February 2021).

### 3.5. Genome-Based Taxonomy and Phylogeny

Species assignment was made by comparing the average nucleotide identity (https://www.ezbiocloud.net/tools/ani), average amino acid identity (http://enve-omics.ce.gatech.edu/aai), in silico genome-to-genome distance similarity, and G+C content (http://ggdc.dsmz.de/ggdc.php#) [29,30] against the reference *B. fragilis* NCTC 9343T (NC_003228.3) genome and the representative *B. fragilis* YCH46 (NC_006347.1) genome [25,55]. For taxonomic and phylogenetic analyses, the genome BLAST distance phylogeny method was used with 186 available *B. fragilis* genomes (13 April 2020); this was performed using the Type Strain Genome Server (TYGS) (open-source in https://tygs.dsmz.de) [31]. Genomes were also subjected to core-genome typing (cgMLST) using chewBBACA v. 2.0.17.2 software (https://github.com/B-UMMI/chewBBACA) [32]. Loci corresponding to potentially complete coding sequences (CDS) with a copy number of one, but which were present in 95% of the studied *B. fragilis* strains, were used in subsequent phylogenetic analysis; GrapeTree v. 2.0 software (https://github.com/achtman-lab/GrapeTree) was used to visualise the results [56] (accessed on 4 December 2020).

The Whole Genome Shotgun project PRJNA656918 was deposited at DDBJ/ENA/GenBank under accessions JACLQC000000000 and JADDIJ000000000.

## Figures and Tables

**Table 1:** Antimicrobial susceptibility, resistant genes, and IS elements identified in the two *cfi*A-positive *B. fragilis* strains resistant to meropenem-EDTA.

Strain	Antimicrobial Agent	MIC (mg/L) ^1^	Interpretation ^2^ EUCAST/CLSI	Antimicrobial Resistance (AMR) Gene (Resistance Mechanism)	% Identity ^3^ (% Length)	IS ^4^ Element
CNM20180471						
	Penicillin Amoxicillin-clavunalate Piperacillin/tazobactam	>32 >256 >256	R/R R/R R/R			
	Imipenem Imipenem+EDTA ^5^ Meropenem Meropenem+EDTA	>32 1 >32 >2	R/R MBL-positive ^6^ R/R MBL-negative	*cfi*A14b ^7^, metallo-β-lactamase (antibiotic inactivation)	100 (100)	
	Amikacin Gentamicin Tobramycin	>32 >32 >32	NA ^8^ NA NA	*aad*S, aminoglycoside 6-adenylyltransferase *aac*(3’), *N*-acetyltransferase (antibiotic inactivation)	100 (100) 99.7 (100)	
	Erythromycin Clindamycin	>256 >256	NA/NA R/R	*erm*F, 23S ribosomal RNA methyltransferase *lnu*(AN2), lincosamide nucleotidyltransferase *vat*A, virginiamycin A acetyltransferase (antibiotic target alteration) *mef*(En2)^,^, major facilitator superfamily (antibiotic efflux pump)	97.7 (100) 100 (100) 99.2(100)	ISBaov1
	Tetracycline Tigecycline	8 0.5	NA NA	*tet*X, tetracycline-inactivating monooxygenase (antibiotic inactivation) *tet*Q, tetracycline-resistant ribosomal protection protein (antibiotic target protection)	100 (100) 96.4 (97.5)	
	Metronidazole	0.38	S/S			
	Chloramphenicol	ND ^8^	NA	*cat*, chloramphenicol acetyltransferase (antibiotic target alteration)	95.77 (100)	
	Linezolid	1	NA			
	Ciprofloxacin Moxifloxacin	>32 >32	NA/NA NA/R	*bex*A ^9^ *bex*B, multidrug efflux MATE transporter (antibiotic efflux)	98.2 (100) 83.2 (100)	
	Quaternary ammonium compounds	-		*qac*E, quaternary ammonium compound resistance (antibiotic efflux)	95.4 (100)	
CNM20200206						
	Penicillin Amoxicillin-clavunalate Piperacillin/tazobactam	>32 >256 >256	R/R R/R R/R			
	Imipenem Imipenem+EDTA Meropenem Meropenem+EDTA	>32 <1 >32 >2	R/R MBL-positive R/R MBL-negative	New *cfi*A28, metallo-β-lactamase (antibiotic inactivation)		
	Amikacin Gentamicin Tobramycin	>32 >32 >32	NA NA NA	*aac*(3´), *N*-acetyltransferase (antibiotic inactivation)	100 (100)	
	Erythromycin Clindamycin	>256 >256	NA/NA R/R	*vat*A, virginiamycin A acetyltransferase (antibiotic target alteration)	100 (100)	
	Tetracycline Tigecycline	16 6	NA NA	*tet*Q, tetracycline-resistant ribosomal protection protein (antibiotic target protection)	96.41 (97.5)	
	Metronidazole	0.5	S /S			
	Chloramphenicol	ND	NA	*cat,* chloramphenicol acetyltransferase (antibiotic target alteration)	95.77 (100)	
	Linezolid	1.5	NA			
	Ciprofloxacin Moxifloxacin	>32 4	R/R NA/I	*bex*A *bex*B, multidrug efflux MATE transporter (antibiotic efflux)	83.8 (100) 98.0 (100)	

^1^ Minimun inhibitory concentration (MIC) values expressed in mg/L. ^2^ Interpretation according to European Committee on Antimicrobial Susceptibility Testing (EUCAST) and Clinical Laboratory Standards Institute (CLSI) breakpoints. ^3^ Percentage of identity of matching region and percentage length of reference sequence. ^4^ Insertion sequence (IS) element. ^5^ EDTA—ethylene diamine tetra-acetic. ^6^ MBL—metallo-β-lactamase. ^7^ With respect to *cfi*A14b (GenBank accession no. KT318729.1). ^8^ NA—not available; ND—not determined. ^9^ With respect to *bex*A (GenBank accession no. AB067769.1) and *bex*B (GenBank accession no. AY375536.1).

## Data Availability

The Whole Genome Shotgun project PRJNA656918 was deposited at DDBJ/ENA/GenBank under accessions JACLQC000000000 and JADDIJ000000000.

## Supplementary Materials

The following are available online at https://www.mdpi.com/2079-6382/10/3/304/s1. Figure S1. Phenotypic detection of *cfi*A in *B. fragilis* CNM20180471 with the meropenem-EDTA (left, negative) and imipenem–EDTA (right, positive) double-ended E-tests. Figure S2: 16S rRNA sequence-based phylogenetic tree constructed using FastME v. 2.1.6.1 software (which calculates genome BLAST distance phylogeny distances; the branch lengths are scaled in terms of the GBDP distance formula). The numbers above the branches are GBDP pseudo-bootstrap support values (all are >60%; 100 replicates); average branch support = 21.3%. The trees were rooted at the midpoint. The results were provided by the Type Strain Genome Server (TYGS, https://tygs.dsmz.de (accessed on 13 October 2020)). The same color code was used in the chewBBACA analysis (Table S1). IS elements and plasmid identities of the two *cfi*A-positive *B. fragilis* strains resistant to meropenem-EDTA. Table S2: Comparison of the genome sequences of *B. fragilis* CNM20180471 and CNM20200206 and other strains against the genome of the *B. fragilis* NCTC 9343T (GCF_000025985.1) type strain and the *B. fragilis* YCH46 (GCF_000009925.1) representative genome, in terms of ANI, AAI, in silico genome-to-genome distance similarity (GGDH; DDH-estimate), and differences in G+C content.
