# Supplementary material for: Genomic Background and Phylogeny of cfiA-Positive Bacteroides fragilis Strains Resistant to Meropenem-EDTA
Source: Antibiotics (Basel). 2021 Mar 16;10(3):304. doi: 10.3390/antibiotics10030304 (PMC8001070; doi:10.3390/antibiotics10030304)
Supplement: Supplementary file 1 [file antibiotics-10-00304-s001.zip › svaldezate_Table S2.docx]

**Table S2.** Comparison of the genome sequences of *B. fragilis* CNM20180471, CNM20200206 and other strains against the genome of the *B. fragilis* NCTC 9343^T^ (GCF_000025985.1) type strain and the *B. fragilis* YCH46 (GCF_000009925.1) representative genome, in terms of ANI, AAI, *in silico* genome-to-genome distance similarity (GGDH; DDH-estimate), and difference in G+C content.

| **Strain identification**  **(assembly no.)** | **ANI**^1-3^ **(% coverage) [≥95%]** | | | **AAI**^1,2,4^ **(SD, from no. of proteins) [≥95%]** | **DDH-estimate^1,2,5^**  **difference in G+C [>70%, <1%]** |
| --- | --- | --- | --- | --- | --- |
| Determination with respect to | **NCTC 9343**  *reference* | **YCH46**  *representative* | **20180471**  *studied strain* | **NCTC 9343** | **NCTC 9343** |
| CNM20180471 *(studied strain)* | *86.96 (50.45)* | *87.12 (50.10)* | 100 (100) | *90.07% (14.03%, 3277)* | *32.80% [30.3 - 35.3%], 0.32* |
| CNM20200206 (studied strain) | *87.23* (50.86) | *87.00 (52.22)* | 98.54 (59.42) | *90.29% (14.02%, 3297)* | *33.10% [30.6 - 35.6%],* 0.53 |
| *Central node* |  |  |  |  |  |
| *NCTC 9343 (GCF_000025985.1)* | 100 (100) | 98.94 (63.48) | *86.96 (50.45)* | 100.00% (0.00%, 4392) | 100.00% [100 - NaN%], 0.00 |
| *CCUG4856T (GCF_005706655.1)^#^* | 99.9 (83.84) | 98.90 (63.87) | *86.89 (53.40)* | 99.97% (1.28%, 4139) | 100.00% [100 - 100%], 0.00 |
| *Selected nodes* (clockwise) |  |  |  |  |  |
| *4g8B (GCF_001373095.1)^7^* | *99.04 (70.34)* | *99.12 (69.32)* | *86.77 (44.90)* | *98.02% (8.70%, 3205)* | *93.90% [92.1 - 95.3%], 1.78 d.s.^6^* |
| OM06-30AC (GCF_003438775.1) | 98.92 (66.57) | 98.94 (67.97) | *86.88 (54.78)* | 97.67% (9.42%,3780) | 91.60% [89.4 - 93.3%], 0.25 |
| GCF_007896745.1_HAP130N_2B | 99.14 (63.97) | 99.10 (66.62) | *86.83 (53.25)* | 97.67% (9.79%, 3781) | 92.70% [90.8 - 94.3%], |
| 143 (GCF_008086695.1) | 99.11 (60.68) | 99.06 (66.85) | 87.03 (54.27) | 97.85% (9.19%, 3806) | 92.80% [90.8 - 94.4%], 0.01 |
| OF05-11AC (GCF_003439675.1) | 98.85 (63.01) | 98.85 (63.01) | *86.74 (51.35)* | 97.64% (9.25%, 3826) | 91.40% [89.3 - 93.2%], 0.42 |
| Bfra_078320_1 (GCF_002811085.1) | 99.00 (66.70) | 99.13 (65.43) | *86.65 (52.55)* | 98.03% (8.33%, 3710) | 92.50% [90.5 - 94.1%], 0.18 |
| Gb10071F10 (GCF_000598685.2) | 99.02 (61.13) | 98.90 (60.31) | *86.89 (50.16)* | 97.92% (8.82%, 3809) | 91.70% [89.6 - 93.4%], 0.13 |
| am_0171 (GCF_004167855.1) | 99.17 (60.93) | 98.92 (64.03) | *86.75 (50.70)* | 97.31% (10.82%, 3773) | 93.30% [91.4 - 94.8%], 0.17 |
| *YCH46 (GCF_000009925.1)* | *98.94 (63.48)* | *100 (100)* | *87.12 (50.10)* | *97.21% (10.49%, 3628)* | *92.60% [90.6 - 94.2%], 0.09* |
| BFR_KZ02 (GCA_004798515.1) | 99.19 (63.05) | 99.18 (59.30) | *86.82 (52.84)* | 97.24% (11.07%, 3544) | 92.60% [90.6 - 94.2%],  0.07 |
| J-143-4gbfJ1434 (GCF_000598525.1) | 99.14 (59.49) | 99.04 (63.85) | *86.80 (48.02)* | 97.77% (9.38%, 3684) | 92.80% [90.9 - 94.4%],  0.02 |
| CL05T00C42 (GCF_000273765.1) | 98.85 (58.50) | 98.79 (59.59) | *86.84 (52.23)* | 96.96% (11.13%, 3605) | 89.70% [87.4 - 91.7%],  0.37 |
| 20793-3 (GCF_001699855.1) | 98.85 (62.74) | 98.71 (61.48) | *87.11 (50.89)* | 96.96% (11.46%, 3465) | 90.70% [88.4 - 92.5%],  0.06 |
| AM31-13AC (GCF_003469305.1) | 98.76 (88.32) | 98.81 (62.78) | *87.02 (54.18)* | 97.18% (10.19%, 3643) | 89.90% [87.6 - 91.8%], 0.31 |
| OM02-11 (GCF_003439505.1) | 98.86 (65.92) | 98.75 (61.19) | *86.91 (52.45)* | 97.16% (10.59, 3562) | 91.6 (0.29)  80.00% [76.6 - 83%], 0.26 |
| *Smaller cluster* |  |  |  |  |  |
| *AF14-14AC (GCF_003465265.1) ^#^* | *87.03 (52.65)* | *87.61 (55.01)* | *98.41 (60.97)* | *90.37% (13.70%, 3338)* | *32.90% [30.4 - 35.4%], 0.18* |
| AF14-26 (GCF_003464635.1) | 87.09 (50.51) | *87.62 (56.00)* | 98.48 (58.87) | *90.36% (13.73%, 3341)* | *32.90% [30.4 - 35.4%], 0.18* |
| *BF8 (GCF_001695355.1)* | *87.11 (52.17)* | *87.65 (54.19)* | *98.23 (61.42)* | *90.10% (13.87%, 3346)* | *32.90% [30.5 - 35.4%], 0.23* |
| HMW_616 (GCF_000297755.1) | *87.91 (51.43)* | *87.60 (52.03)* | 97.86 (55.35) | *91.04% (12.88%, 3421)* | *34.90% [32.5 - 37.4%], 0.25* |
| OF01-1 (GCF_003463545.1) | *87.47 (55.78)* | *87.59 (53.72)* | 97.89 (58.99) | *90.92% (12.95%, 3393)* | *34.20% [31.7 - 36.7%], 0.31* |
| Q1F2 (GCF_002849695.1) | *87.22 (52.47)* | *87.18 (52.34)* | 98.22 (59.53) | *90.85% (12.78%, 3350)* | *33.50% [31.1 - 36%], 0.26* |
| O21 (GCF_001693695.1) | *87.11 (52.89)* | *87.24 (52.34)* | 98.29 (59.63) | *90.41% (13.29%, 3310)* | *32.90% [30.5 - 35.4%], 0.28* |
| 3_1_1 (GCF_000157015.1) | *87.08 (49.59)* | *87.58 (51.66)* | 98.43 (56.52) | *90.33% (13.59%, 3345)* | *33.00% [30.5 - 35.5%], 0.51* |
| *HMW_610 (GCF_000297695.1) ^#^* | *87.91 (51.43)* | *87.18 (55.32)* | *98.82 (62.86)* | *89.97% (14.31%, 3412)* | *48.40% [45.3 - 51.4%], 0.20* |
| GCF_000724665.2_DCMOUH0018B | *86.98 (48.59)* | *86.55 (50.84)* | 98.59 (59.33) | *90.60% (12.87%, 3327)* | *32.60% [30.2 - 35.1%], 0.23* |
| DCMOUH0085B (GCF_000724815.2) | *86.93 (50.60)* | *87.16 (50.42)* | 98.68 (64.10) | *89.93% (14.10%, 3375)* | *32.60% [30.2 - 35.1%], 0.49* |
| DCMSKEJBY0001B (GCF_000710365.2) | *87.29 (50.00)* | *87.29 (51.99)* | 98.68 (63.55) | *90.77% (12.67%, 3432)* | *33.10% [30.7 - 35.6%], 0.34* |
| DCMOUH0017B (GCF_000710375.2) | *87.45 (52.31)* | *87.50 (51.66)* | 98.50 (61.80) | *90.94% (12.71%, 3448)* | *33.70% [31.3 - 36.2%], 0.29* |
| AM18-6 (GCF_003471465.1) | *87.43 (50.02)* | *87.73 (50.8)* | 98.58 (61.64) | *90.59% (13.21%,3436)* | *49.50% [46.4 - 52.5%], 0.22* |
| MGYG-HGUT-00236 (GCF_902364655.1) | *87.65 (52.81)* | *87.27(53.29)* | 98.56 (51.87) | *90.63% (13.65%, 3473)* | *34.00% [31.6 - 36.6%], 0.28* |
| DCMOUH0067B (GCF_000724805.2) | *86.89 (50.83)* | *86.79 (49.51)* | 98.59 (62.81) | *90.62% (12.90%, 3346)* | *32.70% [30.3 - 35.2%], 0.73* |
| OM04-9BH (GCF_003439065.1) | *86.99 (52.07)* | *87.22 (52.35)* | 98.58 (65.96) | *90.26% (13.61%, 3409)* | *33.00% [30.6 - 35.5%], 0.41* |
| AM18-6 (GCF_003471465.1) | *87.43 (50.02)* | *87.73 (50.08)* | 98.58 (61.64) | *90.59% (13.21%, 3436)* | *33.70% [31.3 - 36.2%], 0.22* |
| Closely related *Bacteroides* species |  |  |  |  |  |
| *B. finegoldii* DSM 17565 (GCF_000156195.1) | *75.23 (24.75)* | *76.12 (26.29)* | 75.36 (24.51) | *72.19% (20.75%, 2355)* | *21.10% [18.8 - 23.5%], 0.18* |
| *B. xylanisolvens* ASM654696v1 (GCF_006546965.1) | *75.23 (20.45)* | *77.00 (22.80)* | 75.66 (26.43) | *72.42% (20.45%, 2613)* | *22.70% [20.4 - 25.2%], 1.30 d.s.* |
| *B. ovatus* ATCC 8483 (GCF_001314995.1) | *74.98 (20.56)* | *74.91 (22.11)* | 75.01 (27.76) | *72.01% (20.24%, 2606)* | *21.10% [18.9 - 23.6%], 1.24 d.s.* |

^1^The reference breakpoints for assigning membership to a specific species in terms of average nucleotide identity (ANI), average amino acid identity (AAI), *in silico* genome-to-genome distance similarity (GGDH; DDH-estimate), and difference in G + C content, are indicated in brackets in the column headings. ^2^ Values lower than the reference breakpoints - suggestive of a distinct species - are indicated in italics. ^3^ANI and coverage were determined using the EzBioCloud platform (https://www.ezbiocloud.net/tools/ani), ^4^AAI at the Kostas Laboratory (http://enve-omics.ce.gatech.edu/aai), and the ^5^DDH-estimate and the difference in genomic G + C content using the DSMZ platform ([https://ggdc.dsmz.de/ggdc.php#](https://ggdc.dsmz.de/ggdc.php)). ^6^The interpretation is either 'distinct' (d.s), or 'distinct or same species'. ^7^In italics, strains and genomes occupying reference nodes: CCUG4856T (GCF_005706655.1) at the central node, 4g8B (GCF_001373095.1), AF14-14AC (GCF_003465265.1) at intermediate nodes, and HMW_610 (GCF_000297695.1) at the distal node.
