# Supplementary material for: Genomic Background and Phylogeny of cfiA-Positive Bacteroides fragilis Strains Resistant to Meropenem-EDTA
Source: Antibiotics (Basel). 2021 Mar 16;10(3):304. doi: 10.3390/antibiotics10030304 (PMC8001070; doi:10.3390/antibiotics10030304)
Supplement: Supplementary file 1 [file antibiotics-10-00304-s001.zip › svaldezate_Figure S1.pptx]

## Slide 1
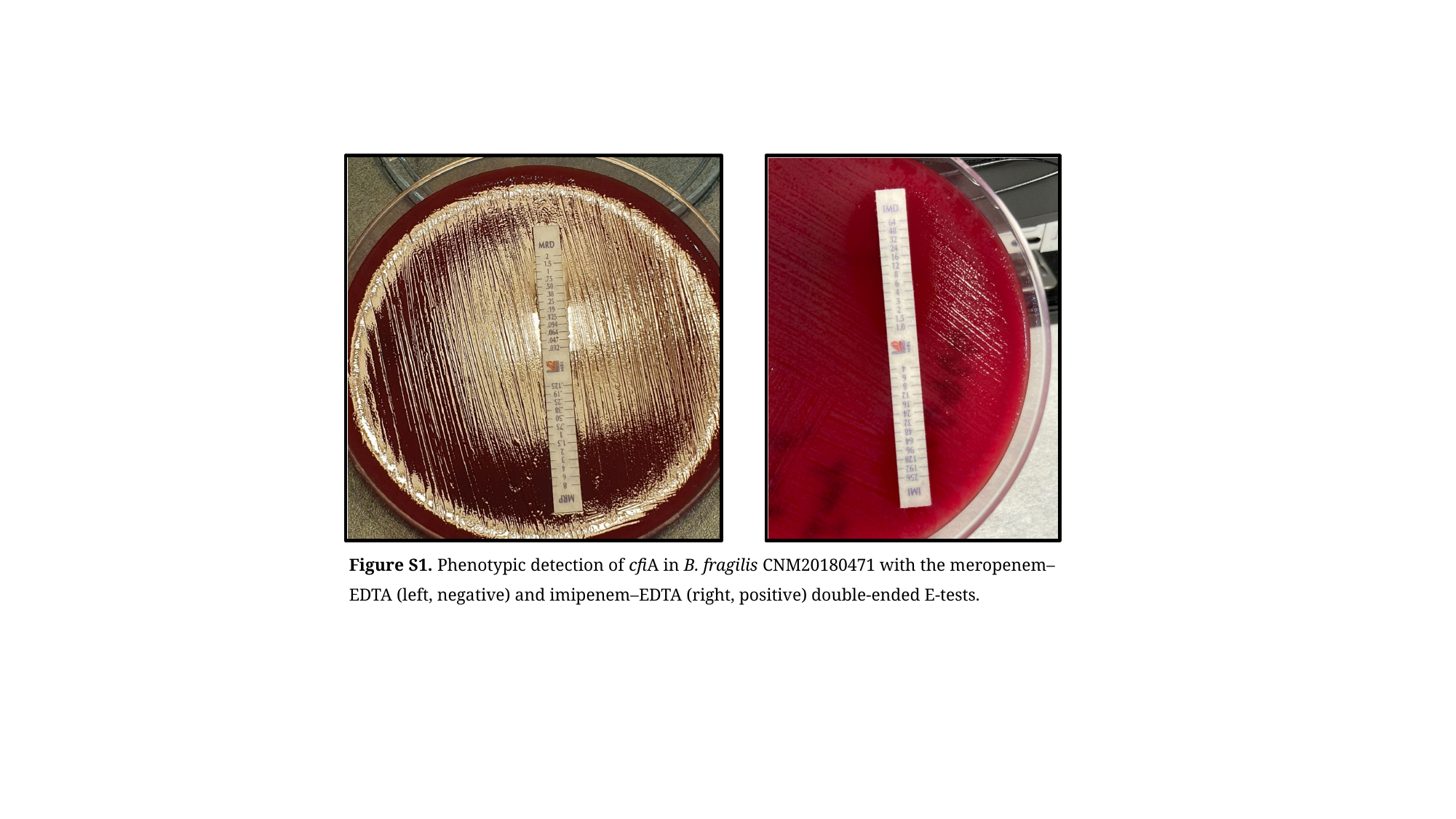

Figure S1. Phenotypic detection of cfiA in B. fragilis CNM20180471 with the meropenem–EDTA (left, negative) and imipenem–EDTA (right, positive) double-ended E-tests.
