# Supplementary material for: Genomic Background and Phylogeny of cfiA-Positive Bacteroides fragilis Strains Resistant to Meropenem-EDTA
Source: Antibiotics (Basel). 2021 Mar 16;10(3):304. doi: 10.3390/antibiotics10030304 (PMC8001070; doi:10.3390/antibiotics10030304)
Supplement: Supplementary file 1 [file antibiotics-10-00304-s001.zip › svaldezate_Figure S2.pptx]

## Slide 1
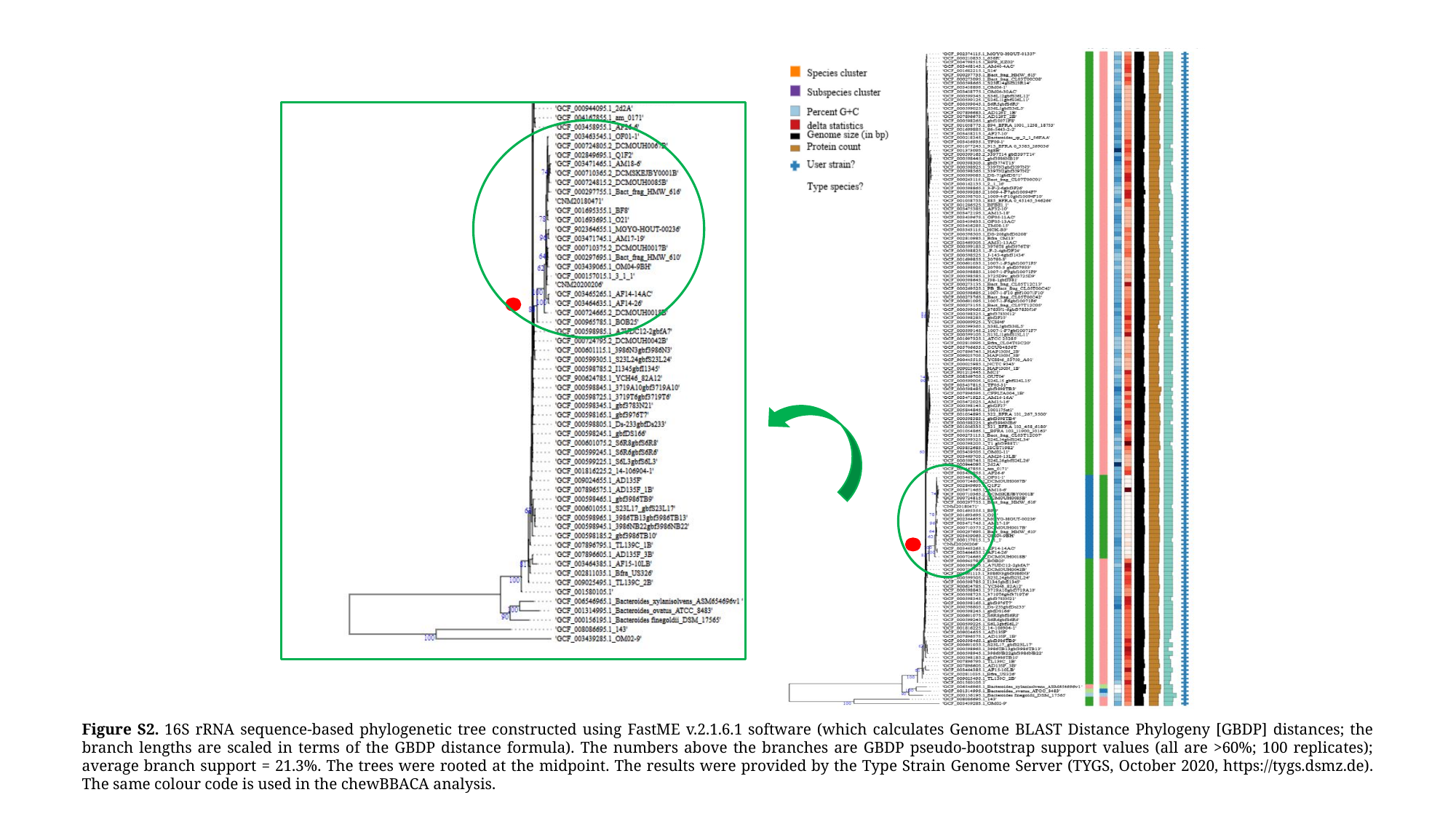

Figure S2. 16S rRNA sequence-based phylogenetic tree constructed using FastME v.2.1.6.1 software (which calculates Genome BLAST Distance Phylogeny [GBDP] distances; the branch lengths are scaled in terms of the GBDP distance formula). The numbers above the branches are GBDP pseudo-bootstrap support values (all are >60%; 100 replicates); average branch support = 21.3%. The trees were rooted at the midpoint. The results were provided by the Type Strain Genome Server (TYGS, October 2020, https://tygs.dsmz.de). The same colour code is used in the chewBBACA analysis.
