# Supplementary material for: Genomic Background and Phylogeny of cfiA-Positive Bacteroides fragilis Strains Resistant to Meropenem-EDTA
Source: Antibiotics (Basel). 2021 Mar 16;10(3):304. doi: 10.3390/antibiotics10030304 (PMC8001070; doi:10.3390/antibiotics10030304)
Supplement: Supplementary file 1 [file antibiotics-10-00304-s001.zip › svaldezate_Table S1.docx]

**Table S1.** *IS* elements and plasmid identities of the two *cfi*A-positive *B. fragilis* strains resistant to meropenem–EDTA.

| **Strain** | **IS elements** | **Copy number** | **Identity percentage**  **(Length coverage percentage)** | **Plasmids**  **(GenBank accession no.)** | **Size**  **(kb)** | **Identity**  **percentage** |
| --- | --- | --- | --- | --- | --- | --- |
|  |  |  |  |  |  |  |
|  |  |  |  |  |  |  |
| CNM20180471 |  |  |  |  |  |  |
|  |  |  |  |  |  |  |
|  | *IS*612B  *IS*Baov1  *IS*Bf3 | 1  1  1, 2 partial | 100% (100)  100% (100)  99.8% (95.6-100) | Q1F2-p1 (NZ_CP018938.1)  pBFUK1 (NC_019534.1)  p2-F9-2 (AP022662.1) | ~4.5  ~13  ~0.8 | 99.12%  95.01%  92.64% |
|  |  |  |  |  |  |  |
|  | *IS*Bf5  *IS*Bf9 | 1  3 | 99.7% (96.1)  86-99% (30-71) |  |  |  |
|  |  |  |  |  |  |  |
|  |  |  |  |  |  |  |
| CNM20200206 |  |  |  |  |  |  |
|  | *IS*4351  *IS*Bf3  *IS*Bf9 | 1  1  1 | 100% (100)  100% (100)  99% (71) | Q1F2-p1 (NZ_CP018938.1)  pBFUK1 (NC_019534.1)  pBFY46 (NC_006297.1) | ~4.5  ~13  ~34 | 98.42%  94.09%  86.45 |
|  |  |  |  |  |  |  |
|  |  |  |  |  |  |  |
